# A Comprehensive Review on Anti-Inflammatory Response of Flavonoids in Experimentally-Induced Epileptic Seizures

**DOI:** 10.3390/brainsci13010102

**Published:** 2023-01-05

**Authors:** Shyam Sunder Rabidas, Chandra Prakash, Jyoti Tyagi, Jyoti Suryavanshi, Pavan Kumar, Jaydeep Bhattacharya, Deepak Sharma

**Affiliations:** 1 Neurobiology Laboratory, School of Life Sciences, Jawaharlal Nehru University, New Delhi 110067, India; 2 Nanobiotechnology Laboratory, School of Biotechnology, Jawaharlal Nehru University, New Delhi 110067, India; 3 Department of Anatomy and Cell Biology, College of Medicine, The University of Illinois at Chicago, Chicago, IL 60607, USA

**Keywords:** epilepsy, seizures, inflammation, anti-inflammatory response, flavonoids

## Abstract

Flavonoids, a group of natural compounds with phenolic structure, are becoming popular as alternative medicines obtained from plants. These compounds are reported to have various pharmacological properties, including attenuation of inflammatory responses in multiple health issues. Epilepsy is a disorder of the central nervous system implicated with the activation of the inflammatory cascade in the brain. The aim of the present study was to summarize the role of various neuroinflammatory mediators in the onset and progression of epilepsy, and, thereafter, to discuss the flavonoids and their classes, including their biological properties. Further, we highlighted the modulation of anti-inflammatory responses achieved by these substances in different forms of epilepsy, as evident from preclinical studies executed on multiple epilepsy models. Overall, the review summarizes the available evidence of the anti-inflammatory potential of various flavonoids in epilepsy.

## 1. Introduction

Epilepsy is an enduring brain disorder characterized by the prevalence of spontaneous and recurrent seizures (SRS). There are numerous types of epilepsies, some of which are influenced by genetic predispositions, some of which are brought on by brain damage, and some of which have an unknown underlying cause [1]. Epileptic seizures are the consequences of an unsynchronized and excessive electrical activity of a group of neurons. The spontaneous electrical discharges during seizures jeopardize the normal brain’s electrical activity and can result in odd sensations or involuntary motions of various body parts [2]. People with epilepsy have a variety of psychological and social difficulties in daily life, including anxiety, sadness, sleep problems, emergency seizures, problems with thinking and memory, a lack of self-confidence, and poor social skills [3,4]. Recent estimates suggest that epilepsy affects almost 70 million individuals globally. The prevalence of epilepsy affects people worldwide, where more than 80% of cases are from low and middle-income countries [5].

The etiology of epilepsy seems to be influenced by several factors, including aging, genetic mutation, central nervous system (CNS) homeostasis, and brain insults, viz. oxidative stress, inflammation, and traumatic brain injury [6]. However, the pathophysiological mechanisms responsible for the onset and recurrence of epileptic seizures, pathological changes, and associated comorbidities remain largely unknown. The dynamic and neurophysiological changes of the brain accountable for ictogenesis can be studied in animal models of genetic and acquired epilepsy [7,8]. The neuroinflammatory pathways have been linked with epileptogenesis and may be the target of disease-modifying therapies [9,10]. Moreover, evidence from human studies has demonstrated the involvement of neuroinflammation in the inception and development of various forms of epilepsy [11]. Thus, the evidence suggests that inflammation within the brain is intricated with the recurrence and precipitation of seizures. Moreover, repetitive seizures have also been reported to induce inflammatory mediators, which may increase brain excitability and neuronal degeneration [12,13].

Epilepsy can sometimes be efficiently treated and cured with the anti-seizure medications (ASMs) already on the market. However, these medications also have at least two significant drawbacks: First, 30% of patients have inadequate seizure control and develop intractable conditions even with excellent ASMs therapy. Second, because these drugs must be taken continuously to prevent seizures, they may have deleterious effects on cognitive development, as they work as general CNS depressants [14]. Thus, for the treatment of refractory epilepsy, novel therapeutic compounds with minimal adverse effects must be developed.

Polyphenols are plant constituents that exist as secondary metabolites and are stored in specialized tissues and vacuoles [15]. Some essential polyphenols are lignans, flavonoids, stiblins, phenolic alcohols, and phenolic acids, which are known to have several health benefits. They are potent antioxidants that are effective against oxidative stress-associated diseases and can alter various cell signaling pathways [16]. These compounds can interfere with inflammatory functions and are highly recommended to prevent inflammatory diseases [17,18,19].

Among the polyphenolic substances, flavonoids exhibit potent anti-inflammatory and antioxidant properties [20,21]. Numerous studies provide evidence that neuroprotective effects of flavonoids are accompanied by their anti-inflammatory properties through various mechanisms [22,23,24]. A growing body of evidence from preclinical studies has also demonstrated that numerous flavonoids exhibit anti-epileptic properties, which also seem to be contributed by their anti-inflammatory functions.

The aim of the present study was to highlight the current understanding of the potential anti-seizure action of flavonoids in diverse epilepsy models, with particular emphasis on the immunomodulatory effects of these compounds in the epileptic brain. Most of the paper comprises experimental investigations executed on animal models of epilepsy, and it details flavonoids’ anti-seizure and immunomodulatory properties.

## 2. Neuroinflammation in Epilepsy

The inflammation process affecting nervous tissues is known as neuroinflammation, and it can be brought on by several exogenous or endogenous sources. Numerous conditions, including infection, toxic insult, autoimmune diseases, aging, traumatic brain injury, and spinal cord injury, can trigger neuroinflammation [25,26,27]. The primary mechanism of neuroinflammation is the activation of microglia and astrocytes by cytokines and chemokines [26]. Interleukins (ILs) are a group of cytokines expressed and released by leukocytes and other body cells. At least 40 different types of ILs are known to be linked with neuroinflammation [27]. Continuous microglial activation triggers the recruitment of peripheral immune cells, including macrophages, B and T lymphocytes, and other immune cells, thus regulating the innate and adaptive immune response [28]. Astrocytes are another type of cells that are activated during neuroinflammation; they have a close connection with the blood-brain barrier (BBB) and are capable of responding to signals released by damaged neurons or reactive microglia [29]. Their contribution to tissue repair can be significant, as seen in the case of glial scars that encourage axonal regeneration [30]. However, persistent, long-lasting insults may activate molecular pathways linked with the inflammatory response in the brain resident cells, leading to an unfavorable process that could impair the CNS [31].

The pathophysiology of epilepsy is complicated and seems to be associated with multiple factors, including neuroinflammation. Numerous published studies have linked neuroinflammation with neurological disorders like epilepsy [31,32,33]. Epilepsy is known to originate from various structural or genetic changes or as secondary consequences of injuries to the brain. It can also have an unknown etiology or be caused by immunological, infectious, or metabolic disorders [34]. Certain chronic inflammatory diseases are known to promote epilepsy and other neurological manifestations. Recent estimates suggest about a five- and four-fold increased risk of epilepsy among children and non-elderly adults suffering from autoimmune disorders [35,36]. Although the altered inflammatory response in damaged neuronal tissue significantly contributes to the onset of epilepsy [32], it is still unclear how this imbalanced regulation of inflammation does so. Moreover, several studies have also shown that recurring epileptic seizures have long-term consequences on neuroinflammation, thus affecting the course and outcome of epilepsy [10,31,33].

**Figure 1:**
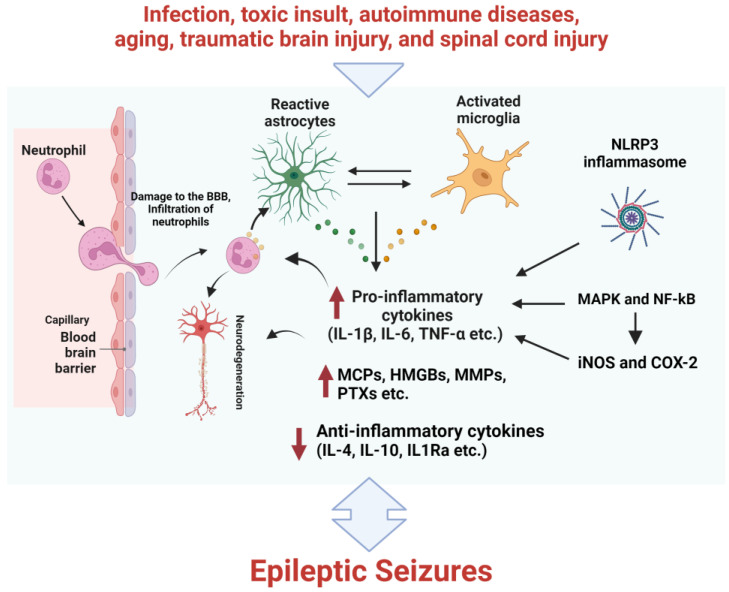
The neuroinflammatory process involved in epilepsy. Pathological insults caused by brain injuries, infections, genetic mutations, etc., stimulates neural cells. These cells produce and release inflammatory mediators in the brain, triggering a cascade of events that resulting in generation of epileptic seizures.

Since the groundbreaking study of Goddard (1967), various mechanisms linking epilepsy to neuroinflammation have been proposed (Figure 1) [37,38]. Interestingly, different experimental models and patients displayed overexpression of the genes of the pro-inflammatory cascade [39]. Cytokines, the small secretory proteins produced by glial cells and neurons, are known to regulate the inflammatory cascades in the epileptic brain [40,41,42]. Interleukin-1β (IL-1β), its receptor (IL-1R), and the receptor antagonist are all known to be altered in the brain of various animal models of epilepsy. The innate immune response is brought on by the activation of toll-like receptors (TLRs), followed by the release of IL-1β from microglial cells [43]. The activation of TLRs is associated with epileptogenesis and a number of other disorders with a secondary epileptic phenotype [44,45,46]. The activation of TLRs can also be amplified by several hyperacetylated molecules, like high mobility group box 1 (HMGB1) [47], along with the development of ictogenesis in human and animal models of chronic epilepsy [48,49].

Reactive microglia may produce excessive levels of nitric oxide (NO) by inducible nitric oxide synthase (iNOS), which can disrupt neuronal mitochondria and activate nicotinamide adenine dinucleotide (NADPH) oxidase, and thus can produce superoxide anion radicals and pro-inflammatory molecules like tumor necrosis factor-α (TNF-α). Moreover, the release of inflammatory cytokines and iNOS is modulated by intrinsic glial pathways, including the mitogen-activated protein kinase (MAPK) and the nuclear factor-кB (NF-кB) signaling pathways [50]. Available evidence demonstrated that the levels of NO, iNOS expression, NF-кB, and MAPK signaling are altered in various epilepsy models. Additional factors that contribute to neuroinflammation, including transforming growth factor-β (TGF-β), cyclo-oxygenase-2 (COX-2), and thrombospondin (TSP-1), have also been documented to alter in epilepsy [51,52,53,54].

It has recently been discovered that members of the pentraxin family (PTXs) proteins are important for inflammatory responses and have been linked to epilepsy [55]. PTX3 is expressed in the brain and released by white blood cells in response to inflammatory signals. Moreover, it can interact with the extracellular matrix, helps to remodel AMPA receptors, and regulates circuit excitability [56,57]. Matrix metalloproteases (MMPs) are calcium-dependent zinc containing endopeptidases and are known to modulate inflammation. The MMP-2 and MMP- 9 are proteases that function extracellularly and control various cellular processes, including neuroinflammation [58]. Studies have shown that neuroinflammation can increase the levels of MMP-2 and MMP-9 in the epileptic brain [58,59]. Changes in the extracellular matrix affect the equilibrium between excitation and inhibition and synaptic plasticity after MMP-9 stimulates the receptor for advanced glycation end-products, which eventually results in the production of numerous cytokines [60]. 

The nucleotide binding and oligomerization domain-like receptor family pyrin domain-containing 3 (NLRP3) inflammasome is a multiprotein complex that activates caspase-1, cleaves pro-IL-1β to form mature IL-1β, and has been identified as a key mediator of IL-1β functions [61,62]. The NLRP3 inflammasome has been shown to regulate innate immunity and inflammation in the CNS [63]. Accumulated evidence from recent studies strongly suggests that NLRP3 inflammasome-mediated inflammation is associated with epilepsy [64,65,66]. 

## 3. Flavonoids

Flavonoids are the most common group of natural polyphenolic compounds in dietary foods and vegetables. These substances share a C6-C3-C6 phenylbenzopyran backbone, which comprises two phenyl rings (A and B) linked by a three-carbon heterocyclic ring (C). Evidence from preclinical research demonstrates flavonoids’ antioxidant, anti-cancer, anti-diabetic, and neuroprotective properties [67,68,69]. Additionally, flavonoids have reduced the risk of neurodegenerative diseases and alleviated neuroinflammation. These compounds can influence inflammatory pathways by inhibiting glial cell activation, cytokine release, NO generation, NADPH oxidase activity, and iNOS expression [21,70]. Flavonoids have been shown to improve human health by influencing key immune system components such as T cells, B cells, mast cells, NK cells, and neutrophils [21,70].

### 3.1. Classification of Flavonoids

**Figure 2:**
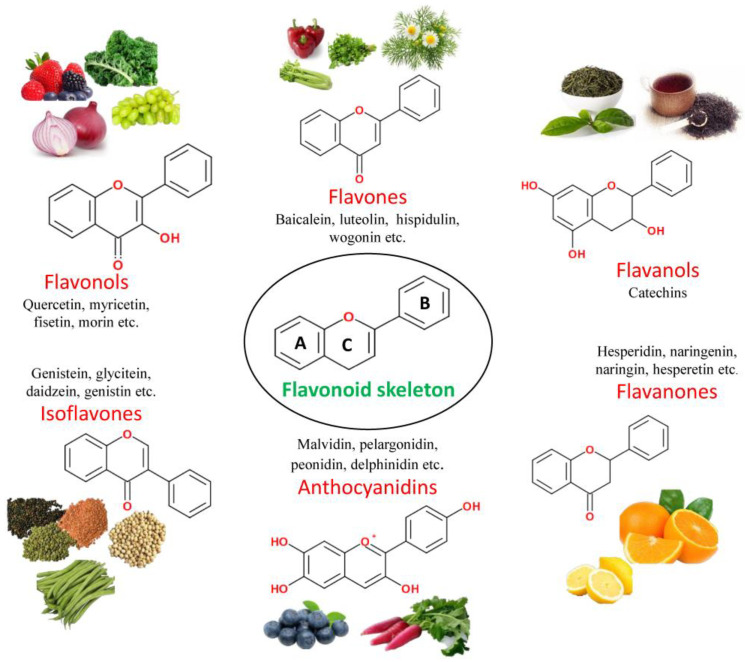
Structure and classification of flavonoids and their dietary sources.

Per their chemical makeup, flavonoids can be characterized into six classes, determined by the degree of C ring oxidation and unsaturation as well as the carbon in the C ring to which the B ring is linked. The benzene and phenyl rings are designated as the A and B rings, respectively, while the oxygen-containing-pyrone ring is referred to as the C ring [71]. Figure 2 represents the major classes of flavonoids, which include flavones, flavonols, flavanones, flavanols, isoflavones and anthocyanidins, as well as their members and sources.

#### 3.1.1. Flavones

One of the major classes of flavonoids is flavones. Compounds in this class have a ketone in position 4 of the C ring and a double bond between positions 2 and 3. Additionally, the majority of flavones found in fruits and vegetables have a hydroxyl group in position 5 of the A ring. The hydroxyl group in other places, like position 7 of the A ring or positions 3 and 4 of the B ring, differs based on the taxonomic classification of the particular fruit or vegetable [72]. Apigenin, luteolin, baicalein, tangeretin, jaceosidin, and eupatilin are all members of this class of flavonoids [70,73].

#### 3.1.2. Flavonols

Flavonols and flavones share a similar structural makeup, except that flavones have an additional hydroxyl group (C-3) that can be glycosylated. Flavonols exhibit a wide range of methylation and hydroxylation as well as distinct glycosylation patterns. When it comes to flavonols, quercetin and kaempferol are the most prevalent in plant foods and continue to be bound to sugar molecules rather than being free [74]. Bioactive compounds like quercetin, kaempferol, fisetin, myricetin, and rutin are important flavonols exhibiting several biological activities, e.g., anti-oxidative, anti-inflammatory, and neuroprotective properties [50,70].

#### 3.1.3. Flavanones

Flavanones, also known as dihydroflavanones, differ from other flavonoids: they lack a double bond between positions 2 and 3 and have a chiral center in position 2 [75]. Flavanones are distinguished from flavones by the absence of a double bond between C-2 and C-3, resulting in a saturated C ring [76]. Among the most researched flavanones are hesperidin, naringenin, eriodictyol, narirutin, and neohesperidin, which have been reported to exert multiple health benefits in oxidative stress and inflammation-linked diseases [70].

#### 3.1.4. Anthocyanidins

Anthocyanidins are sugar-free anthocyanins with an aromatic A ring bound to a heterocyclic C ring with oxygen. The C ring is linked to a third aromatic ring B via a carbon-carbon bond [77]. The absence of a ketone group at position 4 on the C ring distinguishes anthocyanidins from flavonols and flavanones. Anthocyanins are the primary plant pigments, and the pH as well as methylation or acylation at the hydroxyl groups on the A and B rings determines their color [78]. Some of the most significant anthocyanidins are natural flavonoids such as malvidin, pelargonidin, peonidin, cyanidin, petunidin, and delphinidin [21,50].

#### 3.1.5. Isoflavones

Isoflavones are a class of flavonoids with structural similarities to estrogens and 17-β-estradiol that can bind to estrogen receptors [79]. In contrast to other classes, the basic structure of these molecules includes a B ring attached to position 3 of the C ring via aryl migration, rather than position 2 [78]. This class’s important and well-studied bioactive flavonoids include genistein, glycitein, daidzein, daidzin, and genistin [70,79].

#### 3.1.6. Flavanols

Flavanols are a type of flavonoids also known as flavan-3-ols due to the presence of a hydroxyl group at position 3 on the C ring. Moreover, the ketone group at position 4 of the C ring and the C-2 and C-3 do not have a double bond in these molecules [80]. Catechins and epicatechins are the major representatives of this class of flavonoids. The most important catechins are catechin, gallocatechin-3-gallate (GCG), epicatechin (EC), epigallocatechin-3-gallate (EGCG), and epigallocatechin (EGC). All of the aforementioned compounds are created either with or without allyl substituents like epicatechin, catechin, gallocatechin, and epigallocatechin [21,70].

## 4. Flavonoids with Anti-Inflammatory Response in Epilepsy

Flavonoids are a special group of bioactive substances with distinct therapeutic properties. These compounds have been recognized as major constituents of the Indian Traditional Medicine system and the Chinese Herbal Medicine system from time immemorial and are known to work as neuromodulators. The therapeutic potential of these compounds is attributed to their interaction with the immune system and antioxidant effects [81]. A growing body of research has investigated the effectiveness of various flavonoids against diverse forms of epilepsy. Moreover, the flavonoids’ structure is vital and is responsible for their anti-inflammatory action. The positions of the hydroxyl groups are critical to imparting this feature, since they have a planar ring structure with unsaturation at C2-C3. In order to retain their anti-inflammatory effect, the hydroxyl groups at the 3′ and 4′ positions of the B ring of flavonoids are crucial [21]. Thus, it is plausible to speak of the anti-inflammatory response of flavonoids in connection with the treatment of epilepsy. Hereunder, we reviewed the beneficial effect of these flavonoids in epilepsy with particular emphasis on their anti-inflammatory properties (Table 1).

### 4.1. Baicalein

Baicalein (5,6,7-trihydroxyflavone) is a flavone isolated from the roots of *Scutellaria baicalensis* and *Scutellaria lateriflora*. Baicalein has shown beneficial effects against dozens of diseases, including neurological disorders, and inflammation. Its therapeutic efficacy has also been reported in multiple epilepsy models, and it improves seizure propensity and cognitive deficits [84,119]. According to a study by Qian et al. [82], baicalein treatment after the onset of SRS helps temporal lobe epilepsy (TLE) rats to improve cognitive deficits and protects their hippocampal neurons. Additionally, the research has shown that baicalein reduces oxidative stress and inflammation markers (TNF-α and IL-1β) in the sera and hippocampus of TLE rats. Oral administration of baicalein (20, 40 and 80 mg/kg) improved epilepsy symptoms, attenuated microglial proliferation, decreased the expression of Insulin-like growth factor 1 receptor (IGF-1) and inhibited inflammation, as evident from the reduced expression of IL-1β, IL-6, and TNF-α in the brain of pilocarpine-induced epileptic rats [83]. Baicalein (10, 20 and 40 mg/kg) pretreatment for 14 days in tremor rats (TRM) reduced epileptiform activity and improved cognitive impairments. Furthermore, decreased oxidative stress and inflammatory responses were observed, along with changes in the HSP70 and MAPK cascades [84]. Baicalin (50 and 100 mg/kg) showed an anti-seizure effect and improved cognitive dysfunctions in PTZ-induced epileptic rats. Additionally, it decreased the production of IL-1β and IL-6 and activated the TLR4/MYD88/Caspase-3 pathway while reducing neurodegeneration in the CA3 region of the hippocampus [85]. These findings suggest that the anti-epileptic effects of baicalin may be accompanied by a reduction of neuroinflammation and an activation of the TLR4/MYD88/Caspase-3 pathway.

### 4.2. Luteolin

Luteolin (3′,4′,5,7-tetrahydroxyflavone) is a flavone found in various foods and beverages, including pepper, celery, broccoli, thyme, and chamomile tea. This bioactive compound can cross the brain and have various biological effects, including neuroprotection. Lin et al. [86] investigated the anti-inflammatory effect of luteolin in rats with KA-induced seizures. The study showed that luteolin protects neuronal loss, inhibits glial activation, and boosts Akt activation in the hippocampus of KA-injected rats. Pretreatment with luteolin (10 mg/kg i.p.) in PTZ-injected rats showed a reduction in the frequency of seizures, a decrease in iNOS and MMP-2 activity, and an increase in eNOS activity [87]. Together, these studies indicate that suppressing the activation of glial cells, iNOS, and MMP-2 may contribute to the anti-inflammatory response of luteolin in epilepsy.

### 4.3. Hispidulin

Hispidulin (4′,5,7-trihydroxy-6-methoxyflavone) is a flavone that occurs naturally and is an active ingredient in various traditional Chinese medicinal herbs, including Artemisia and Salvia species [120]. Its powerful anti-oxidative, anti-fungal, anti-inflammatory, anti-cancer, and anti-neoplastic capabilities have been proven by several in vitro investigations [121,122,123]. It can cross the BBB, modulates the GABA receptors, and has anti-convulsant characteristics [124]. In a study by Lin et al. [88], it was found that giving hispidulin intraperitoneally (10 and 50 mg/kg) to rats before KA injection reduces the severity of their seizures and the expression of pro-inflammatory cytokines like IL-1β, IL-6, and TNF-α, as well as microglial activation. The study suggests that the anti-seizure effect of hispidulin may be led by its anti-inflammatory properties.

### 4.4. Schaftoside

Schaftoside, also known as apigenin 6-C-glucoside-8-C-arabinoside, is a flavonoid found in many Chinese medicinal herbs, including *Eleusine indica*, *Dendrobium nobile*, *Lysimachia christinae Hance*, and *Capsicum annuum* [125]. Previous research revealed that schaftoside has various pharmacological effects, including anti-inflammatory, anti-melanogenic, and anti-oxidative properties [126]. In zebrafish, schaftoside pretreatment alleviated PTZ-induced seizures by suppressing apoptosis and decreasing the expression of inflammatory cytokines like IL-1β, IL-6, and NF-кB [89]. These results indicate that schaftoside ameliorates seizure progression mainly by an NF-кB mediated inflammatory response and apoptosis.

### 4.5. Vitexin

Vitexin (5,7-dihydroxy-8-methoxyflavone), a c-glycosylated flavone, is an essential constituent of traditional Chinese medicines. This flavonoid is mainly found in passion flowers, bamboo leaves, wheat leaves, pearl millet, mosses, Passiflora, etc. Vitexin has recently gained popularity due to its antioxidant, anti-inflammatory, anti-hyperalgesic, anti-cancer, and neuroprotective properties [127,128]. According to Luo et al. [90], vitexin effectively alleviated spontaneous seizures in neonatal rats with hypoxic ischemia-induced seizures (HINS). Further, the study reported that vitexin decreases neutrophil infiltration and the expression of IL-1β, IL-6, and TNF-α in the rat brain, indicating that the cytokines’ neuroinflammatory response may also be responsible for vitexin’s potential anti-seizure effects.

### 4.6. Wogonin

Wogonin is an O-methylated flavone that can be found in a variety of plants, including the roots of *Scutellaria baicalensis*. Wogonin and its derivatives possess various health benefits, including antiviral, anti-cancer, antioxidant, anti-inflammatory, and neuroprotective properties [129,130]. Recently, Guo et al. [91] investigated the anticonvulsant effect and underlying mechanism of wogonin in KA-induced TLE rats. The study found that wogonin treatment improves cognitive functions and reduces oxidative stress, cellular apoptosis, and inflammatory mediators such as IL-1β, TNF-α, and NF-кB in the hippocampus. Overall, the findings indicate that wogonin’s neuroprotective effect in epilepsy may also be mediated by its immunomodulatory properties.

### 4.7. Rhoifolin

Rhoifolin (apigenin 7-O-neohesperidoside) is a well-known apigenin family tri-substituted flavone. It is found in various foods and plants, including grapefruits, bitter oranges, lemons, grapes, tomatoes, and bananas [131]. Preclinical evidence suggests that rhoifolin has significant biological effects, including antioxidant, anti-inflammatory, anti-arthritic, and anti-cancer properties [132,133,134]. The effectiveness of rhoifolin was evaluated in acquired epilepsy using hippocampal neuronal cultures. The study found that rhoifolin increases cell viability, decreases apoptosis, alleviates oxidative stress, and lowers levels of pro-inflammatory cytokines (IL-1β, IL-6, and TNF-α) along with p-p65, p-Ikb, iNOX, and COX-2 [92]. Thus, the study suggests that the neuroprotective effect of rhoifolin in acquired epilepsy may be attributed to its anti-oxidative and anti-inflammatory potential via inhibition of the NF-кB/iNOS/COX-2 axis.

### 4.8. Amentoflavone

Amentoflavone is a biflavonoid that is created when two apigenin molecules are joined together through oxidation and form a bond at positions C-3 of the hydroxyphenyl ring and C-8 of the chromene ring. This biflavonoid can be isolated and identified in over 120 plants, some of which have been used for hundreds or even thousands of years as traditional folk medicines in different parts of the world [135]. Amentoflavone has a wide range of biological effects, including antioxidant, anti-inflammatory, anti-senescence, anti-tumor effects, and is advantageous against cardiovascular and CNS diseases [135,136,137,138,139,140]. The intragastric administration of amentoflavone (25 mg/kg) to pilocarpine-kindled mice for three days reduced the severity of epileptic seizures and decreased prostaglandin E2 and NF-кB expression, IL-1β and IL-6 production, and neuronal loss and apoptosis in the hippocampus [94]. Similarly, a study by Rong et al. [93] demonstrated that amentoflavone decreases seizure susceptibility, cognitive impairments, and the loss of hippocampus neurons in PTZ-kindled mice. Moreover, amentoflavone reduced inflammatory cytokines (IL-1β, IL-18, and TNF-α) and inhibited NLRP3 inflammasome activation. These findings showed that amentoflavone subdues epileptogenesis, exerts neuroprotective benefits in PTZ-induced kindling mice via suppressing pro-inflammatory molecules and the NLRP3 inflammasome, and modulates the inflammatory process.

### 4.9. Quercetin

Quercetin (3,3′,4′5,7-pentahydroxyflavone) is a widespread flavonol found in fruits, vegetables, seeds, nuts, flowers, bark, and leaves. It is a highly recommended natural component for treating inflammatory and metabolic diseases. A thorough analysis of the literature reveals that quercetin has various pharmacological effects, including antioxidant, anti-inflammatory, neuroprotective, and anti-epileptic actions [141,142,143]. According to a study by Mkhize et al. [95], quercetin has the ability to treat febrile seizures, as it downregulates the expression of several pro-inflammatory cytokines, such as TNF-α, IL-1β, and IL-6 in the presence of prenatal stress. Furthermore, a recent study discovered that quercetin inhibits the production of pro-inflammatory cytokines such as TNF-α and IL-1β and activates NF-кB in the glial cells [96]. Similarly, Ahmed et al. [99] found that quercetin treatment in PTZ epileptic mice reduces the severity of seizures, improves behavioral issues, and repairs cellular damage. The study revealed that quercetin decreases the expression of pro-inflammatory cytokines, e.g., TNF-α, IL-1β, IL-6, and NF-кB, while increasing the expression of anti-inflammatory cytokines, e.g., IL-1Ra, IL-4, and IL-10. Recently, Wu et al. [98] demonstrated that quercetin attenuates later-life seizure susceptibility, anxiety-related behavior, and memory impairments in the rat model of hypoxia-induced neonatal seizure (HINS). Additionally, the study exhibited that quercetin decreases the expression of TNF-α, IL-6 MCP-1, IL-1β, and iNOS in the serum and hippocampus, as well as the protein levels of TLR4, and p-NF-kB p65 in the hippocampus. The effectiveness of quercetin-loaded magnetic nanoparticles was evaluated in PTZ-kindled mice [97]. The research showed that quercetin nanoparticles reduce epileptic seizures and neuronal death in rats by decreasing astrocytic activation. The study also suggested that the anti-seizure effects of quercetin nanoparticles may have been enhanced due to quercetin’s improved bioavailability. These findings suggest that quercetin’s anti-inflammatory response in epilepsy may be aided by inhibiting astrocyte and microglia activation and downregulating NF-кB and pro-inflammatory cytokines, along with upregulating anti-inflammatory molecules.

### 4.10. Rutin

Rutin (quercetin-3-rutinoside), also known as rutoside and sophorin, is an integral derivative of quercetin, which is abundantly present in peaches, oranges, lemons, grapes, apples, berries, nuts, and other fruits and vegetables. This bioactive compound is quercetin and rutinose-containing flavonol glycoside [144]. Accumulating evidence indicates that rutin effectively prevents seizures in animal models of epilepsy [143,145]. A recent study looked into rutin’s possible anti-seizure mechanism in KA-treated rats. The researchers discovered that pretreatment with rutin (50 and 100 mg/kg) for 7 days reduces seizure severity, reverses neuronal loss, and increases glutamate levels in the hippocampus. Rutin also inhibited activation of astrocytes, decreased the protein levels of pro-inflammatory molecules such as IL-1β, IL-6, TNF-α, HMGB1, IL-1R1, and TLR-4, and increased the level of anti-inflammatory molecules IL-10 [100]. Overall, the study found that rutin reduces KA-induced seizures and neuronal loss in rats by suppressing the IL-1R1/TLR4-related neuroinflammatory cascade.

### 4.11. Fisetin

Fisetin (7,3′,4′- flavon-3-ol) is a tetra hydroxy flavone found in a variety of fruits and vegetables, including strawberries, apples, grapes, onions, cucumbers etc. This flavonoid has marked antioxidant activity that makes it a potent therapeutic agent for various health issues [146,147]. Growing evidence suggests the anti-oxidative and neuroprotective effects of fisetin in epilepsy [148,149]. According to Khatoon et al. [101], fisetin reduced myoclonic jerks and seizures in PTZ-induced mice by reducing neuroinflammation and apoptosis, as evident from the decreased expression of HMGB1, TLR-4, IL-1R1, IL-1β, IL-6, and TNF-α in the cortex and hippocampus regions of the brain. Hence, the findings suggest that fisetin’s anti-epileptic potential is implicated in experimental epilepsy and may be accompanied by suppressing the release of pro-inflammatory and apoptotic molecules.

### 4.12. Kaempferol

Kaempferol (3,5,7-trihydroxy-2-(4-hydroxyphenyl)-4H-1-benzopyran-4-one) is a natural flavonol found in fruits and vegetables, including broccoli, apples, strawberries, and beans [150]. Evidence from the available literature suggests that kaempferol has potential therapeutic properties to treat various human diseases including cancer, inflammatory diseases, cardiovascular diseases, neurological diseases etc. [151,152,153,154]. Recently a study by Ahmed et al. [99] found that kaempferol treatment in PTZ epileptic rats attenuates seizure severity, improves behavioral impairments, and restores cellular damage. Further, the study revealed that kaempferol decreases the expression of pro-inflammatory cytokines e.g., TNF-α, IL-1β, IL-6 and NF-кB, while increasing the expression of anti-inflammatory cytokines e.g., IL-1Ra, IL-4 and IL-10. Hence, the anti-epileptic potential of kaempferol may be accompanied by its role in suppressing the pro-inflammatory molecules, while enhancing the anti-inflammatory ones.

### 4.13. Morin

Morin (3,5,7,2′,4′-pentahydroxyflavone) is a yellow flavonol found in various plants, most notably the Moraceae family [155]. Morin has a wide range of pharmacological benefits, including antioxidant and anti-inflammatory properties [156]. The flavonoid’s beneficial effects have been investigated in different neurological disorders, including epilepsy [157,158,159]. The oral administration of morin hydrate to KA-injected mice reduced seizures susceptibility and inhibited granule cell dispersion (GCD) and the activation of the mammalian target of rapamycin complex 1 (mTORC1), along with the restoration of apoptotic proteins and pro-inflammatory molecules (IL-1β, TNF-α, and iNOS) in the hippocampus [103]. Similarly, morin (10 mg/kg, i.p.) reduced seizure severity in PTZ-kindled rats, improved cognitive deficits, and diminished neuronal loss and astrogliosis. The study further showed that the pro-inflammatory cytokines and receptors like TNFR-1, TNF-α, IL-1β, and IL-6 were diminished, and the hippocampal IL-6/p-JAK2/p-STAT3/GFAP cue was significantly decreased [102]. Together, these immunomodulatory effects provide evidence for morin’s promising role in the treatment of epilepsy.

### 4.14. Myricetin

Myricetin (3,3′,4′,4,5,5′,7 hexahydroxyflavone) is another important flavonoid classified as a flavonol [160]. It is a key component of many human meals and beverages, including vegetables, teas, and fruits. It is best known for its iron-chelating, antioxidant, anti-inflammatory, and anti-cancer properties [160,161]. Myricetin supplementation has been proven to have therapeutic effects on a number of nervous system disorders, including cerebral ischemia, Alzheimer’s disease, Parkinson’s disease, epilepsy, and glioblastoma [162]. Myricetin (100 or 200 mg/kg) administered orally for 26 days, prior to each PTZ injection to mice, reduced seizure and mortality rates, downregulated the expression of apoptotic proteins, and restored GABA and glutamate levels. Additionally, it has downregulated the expression of MMP-9 following PTZ-kindling [104], thus suggesting that myricetin’s anti-seizure potential is attributed to its MMP-9-mediated immunomodulatory properties.

### 4.15. Myricitrin

Myricitrin (myricetin-3-O-a-rhamnoside) is a flavonol made up of myricetin linked to an alpha-L-rhamnopyranosyl at position three. The antioxidant activity of myricitrin was demonstrated to be significant, with greater free radical scavenging activity than that of other flavonol rhamnosides or quercetin. This flavonoid is abundant in the dry bark of *Myrica rubra* (Lour.) and exerts many bioactivities, including anti-inflammatory, anti-oxidative, and anti-fibrotic effects [163,164]. According to Keikhaei et al. [105], pretreatment with myricitrin prevented seizures, improved spatial learning and memory, and restored oxidative stress and inflammatory markers (TNF-α) in rats with KA-induced acute and chronic epilepsy. Overall, these findings suggest that myricitrin’s antioxidant and TNF-α-mediated anti-inflammatory potential may help to alleviate seizures in epilepsy.

### 4.16. Galangin

Galangin (3,5,7-Trihydroxy-2-phenyl-4H-1-benzopyran-4-one) is a flavonol found in honey, *Alpinia officinalis*, *Helichrysum aureonitens*, and propolis in substantial concentration. Galangin shows a variety of pharmacological activities, such as anti-cancer, anti-mutagenic, anti-clastogenic, and anti-oxidative properties [165,166,167]. Moreover, numerous studies have exhibited the neuroprotective potential of galangin in various neurological disorders [168,169]. Recently, a study by de Zorzi et al. [106] has exhibited that galangin reduces the risk of seizure severity by regulating multiple neurochemical alterations like activation of microglia and astrocytes in PTZ-induced seizures in mice. Thus, the study gives a hint that galangin’s anti-seizure potential may also be accompanied by its anti-inflammatory properties.

### 4.17. Naringin

Naringin (4′,5,7-trihydroxyflavanone-7-rhamnoglucoside) is a flavone glycoside found in citrus fruits such as grapefruits, pummelos, and sour oranges. Naringenin is one of the active compounds used in Chinese herbal medicine. In recent years, the bioactive compound has attracted the interest of researchers, as it possesses a plethora of biological and pharmacological properties [151]. Naringin is one of the most effective flavonoids for suppressing TNF-α production in PTZ-induced seizures [107,109]. Treatment with naringin in male C57BL/6 mice after KA injection postponed the start of seizures and reduced the frequency of SRS. Additionally, naringin therapy reduced autophagic stress, preserved hippocampal CA1 neurons, and decreased a rise in TNF-α in activated microglia [108]. These findings imply that the anti-autophagic stress and anti-neuroinflammatory properties of naringin may help to prevent epileptic seizures and neuronal death in the hippocampus.

### 4.18. Naringenin

Naringenin (4′5,7-trihydroxyflavanone) is a naringin metabolite found in grapefruit and other citrus fruits. It is a deglycosylated naringin variant and readily crosses the BBB. The flavonoid has demonstrated antioxidant, anti-inflammatory, and neuroprotective properties in experimental models of brain diseases [170]. Naringenin has also been shown to lessen the severity of seizures in pilocarpine and PTZ-induced mouse models; thus, it can be a probable therapeutic agent for epilepsy. Furthermore, Park et al. [110] found that naringenin delayed the onset of seizures and reduced GCD by inhibiting the activation of the mTOR1 in both neurons and reactive astrocytes, as well as reducing the level of pro-inflammatory cytokines like TNF-α and IL-1β in the dentate gyrus of KA-injected mice. Overall, these findings indicate that naringenin may help to prevent epileptic-seizure-induced damage in the hippocampus of the TLE model by mitigating neuroinflammation.

### 4.19. Hesperetin

Hesperetin (4′-methoxy-3′,5,7-trihydroxyflavanone) is a dihydroflavone derived from the breakdown of hesperidin and found in young citrus fruits such as lemons and sweet oranges [171]. Accumulating evidence from preclinical research showed encouraging results for hesperetin in the treatment cancer, cardiovascular and neurodegenerative diseases, and other health issues. Clinical research has confirmed that hesperetin has potential cardioprotective and neuroprotective properties [172]. According to Baradaran et al. [173], hesperetin (50 mg/kg) reduces seizures and oxidative stress in a PTZ-induced seizures model. Furthermore, Kwon et al. [111] discovered that hesperetin reduces GCD and inhibits the expression of the pro-inflammatory molecules produced by activated microglia in the hippocampus of KA-treated mice.

### 4.20. Hesperidin

Hesperidin (3,5,7-trihydroxyflavanone 7-rhamnoglucoside) is a -7-rutinoside of hesperetin found in citrus fruits like lemons, sweet oranges, and grapefruits. It has numerous pharmacological effects, including anti-cancer, anti-inflammatory, anti-hyperlipidemic, anti-hypertensive, diuretic, antiviral, and calcium channel blocking actions [174]. Hesperidin and its aglycone can cross the BBB and exhibit neuroprotective effects in in vitro and in vivo experiments [175]. Recently, Sharma et al. [113] have also explored the possibility that hesperidin therapy improves seizure latency, reduces hyperactive responses in PTZ-mediated seizures in zebrafish larvae, and modulates the expression of BDNF and IL-10. Hesperidin’s affinity for several receptors, including IL-10, was also demonstrated via in-silico research. Hesperidin (100 mg/kg) given to pregnant rats reduced TNF-α, IL-10, and TLR4 protein expression and improved cognition in male offspring with febrile seizures [112]. Overall, these findings suggest that hesperidin has anti-seizure effects, possibly due to its anti-inflammatory properties and TLR4 downregulation.

### 4.21. Silibinin

Silibinin, commonly known as silybin, is a natural flavanone derived from *Silybum marianum* fruits and seeds. It is silymarin’s principal active component and has been demonstrated to provide a range of pharmacological health advantages, including anti-oxidative, anti-inflammatory, anti-cancer, and neuroprotective benefits. According to Kim et al. [115], silibinin therapy decreased the susceptibility to and frequency of SRS in KA-injected mice. The study also showed that silibinin decreases the abnormally high levels of apoptotic and pro-inflammatory molecules (TNF-α and IL-1β) in mice with KA injections. Similarly, Wu et al. [114] found that silibinin has anti-inflammatory and anti-apoptotic effects in the rat model of TLE, and inhibits overexpression of TNF-α, IL-1β, IL-6, caspase-3, cleaved caspase-3, and HIF-1α in the hippocampus. These findings suggest that silibinin’s anti-epileptic and neuroprotective role may be connected with its inhibition of neuroinflammation.

### 4.22. Genistein

Genistein (5,7-dihydroxy-3-(4-hydroxyphenyl)chromen-4-one) is a flavonoid that belongs to the isoflavone class. It is a phytoestrogen derived primarily from legumes like lupines, fava beans, soybeans, kudzu, and Psoralea. Preclinical evidence suggests that genistein has a variety of pharmacological actions, including antioxidant, anti-inflammatory, anti-microbial, angiogenesis, anti-cancer, and neuroprotective properties [176]. The administration of genistein (5 or 10 mg/kg) to PTZ-induced epileptic rats reduced seizure intensity and duration, inhibited astrocytes and microglial activation, and decreased the mRNA and protein expression of p-JAK2, p-STAT3, TNF-α, and IL-1β in the hippocampus [116]. These findings suggest that the anti-seizure effect of genistein may be accompanied by the inhibition of astrogliosis and the JAK2/STAT3 inflammatory pathway.

### 4.23. Catechin

Catechin is a class of flavonoids produced by the plant *Camellia sinensis* var. Sinensis. Growing research has shown that catechin has several preventive properties for the treatment of fever, inflammatory diseases, wounds, and malignancies [177,178]. It is well known to reduce inflammation through the inhibition of NF-кB and pro-inflammatory cytokines [179]. Recently, a study by Ahmed et al. [99] investigated the way that catechin treatment in PTZ epileptic rats attenuates seizure severity, improves behavioral impairments, and restores cellular damage. Further, the study revealed that catechin decreases the expression of pro-inflammatory cytokines, e.g., TNF-α, IL-1β, IL-6, and NF-кB, while increasing the expression of anti-inflammatory cytokines, e.g., IL-1Ra, IL-4, and IL-10. These results indicate that catechin exerts its anti-epileptic potential by modulating pro-and anti-inflammatory cytokines.

### 4.24. Epigallocatechin-3-Gallate

(-)-Epigallocatechin-3-gallate (EGCG) is the major catechin found in green tea. EGCG has been linked to a variety of potential health advantages, such as antioxidant effects, cancer chemoprevention, reduced weight loss, improved cardiovascular health, and protection against ionizing radiation-induced damage to the skin [180,181,182]. EGCG alleviated SRS frequency, cognitive impairment, synaptic dysfunction, and loss of hippocampal neurons, along with the expression of TRL-4, NF-кB, and IL-1β levels in the lithium-pilocarpine treated rats [117]. Similarly, free EGCG and EGCG-loaded PEGylated-PLGA nanoparticles reduced the number and severity of seizures, neuronal death, and glial activation in the hippocampus of KA-injected mice [118]. Together, these studies imply that EGCG can suppress seizures and improve cognitive function and neuronal loss in epilepsy through its anti-inflammatory action.

## 5. Mechanism of Action of Flavonoids in Epilepsy

The anti-inflammatory action of flavonoids is known to be regulated by numerous pathways. The available research focusing on flavonoids and their mechanisms in the context of epilepsy indicated that flavonoids have a distinctive way of modulating of neuroinflammation. Hereunder, we discuss the neuromodulatory mechanism of action of flavonoids in different experimental models of epilepsy.

The anti-inflammatory activity of baicalein was accompanied by the activation of ERK, while inhibiting p-JNK and extracellular signal-regulated kinase like MAPK, and by NF-кB activation in TRM rats [84]. Baicalein has also suppressed the activation of the TLR4/MYD88/caspase-3 signaling pathway in PTZ-kindled rats [85]. Furthermore, it can ameliorate the inflammation by inhibiting IGF1R, a tyrosine kinase receptor that activates PI3K and promotes Akt and mTOR pathways in pilocarpine-induced epilepsy in rats [83]. Similar to baicalein, the anti-inflammatory action of hispidulin was also mediated by suppressing the activation of MAPK pathway [88]. Both NOS and MPPs are important mediators of inflammation, and luteolin interferes with these mediators in PTZ-kindling [87]. Vitexin reduces hypoxia-ischemia-induced seizures by inhibiting neutrophil infiltration and IL-1β, IL-6, and TNF-α expression in rats [90]. Wogonin reduces the level of IL-1β and TNF-α by decreasing the expression of NF-kB in TLE [91]. Rhoifolin was observed to modulate the NF-κB/iNOS/COX-2 signaling cascade in the acquired epilepsy [92]. Amentoflavone influences epileptogenesis and exhibits neuroprotective effect by inhibiting the NLRP3 inflammasome, a mediator of the caspase1/IL-1β cascade of inflammatory process, in PTZ-kindled mice [93]. It can also inhibit NF-кB signal pathway and inflammatory mediators like NO and PGE2, and pro-inflammatory cytokines like IL-1β and IL-6 in pilocarpine-induced epilepsy [94].

Quercetin showed its anti-inflammatory action by suppressing the TLR4/NF-kB signaling pathway in the animal models of experimental epilepsy [98,99]. One of its derivatives, rutin, reduced the release of inflammatory molecules like IL-1β, IL-6, TNF-α, and HMGB1 and inhibited IL-1R1 and TLR4 expression in KA-injected rats [100]. In a study, fisetin was observed to exhibit its anti-inflammatory action by inhibiting the NF-кB/COX2, IL-1R1/TLR4, and AkT/mTOR signaling cascades in PTZ-kindled mice [101]. Morin was found to inhibit neuroinflammation by downregulating the IL-6/p-JAK2/p-STAT3/GFAP cue, as evidenced by the decreased expression of p-JAK2, p-STAT3, and GFAP, along with the other inflammatory mediators [102]. The flavonoid has also prevented GCD and suppressed the mTORC1 pathway in KA-induced seizures [103]. Naringenin was found to inhibit the microglia-derived neuroinflammation in the DG of KA-treated mice, probably by inhibiting GCD and mTORC1 activation [110]. Similarly, hesperetin was shown to alleviate the neuroinflammatory response by suppressing GCD by inhibiting mTORC1 activation [111], while the anti-inflammatory action of hesperidin was accompanied by TLR4 expression [112] and c-fos [113] in experimentally induced seizures. Similar to that of morin and hisperetin, the anti-inflammatory response of silibinin has also been associated with the inhibition of GCD by suppressing mTORC1 activation [115] and HIF-α expression [114] in the hippocampus of epileptic rodents. Genistein can attenuate the activation of microglia and astrocytes and combat inflammation by suppressing the JAK2/STAT3 pathway [116]. The anti-inflammatory potential of EGCG, observed on lithium-pilocarpine-induced epilepsy, is mediated by inhibiting the TLR4/NF-kB signaling pathway [117]. Hence, the available evidence from preclinical data suggests that the anti-inflammatory action of various flavonoids may have been contributed by the modulation of signaling pathways like TLR4/MYD88/caspase-3 signaling, iNOS/NF-кB/COX-2, JAK2/STAT3, IL-1R1/TLR4 and AkT/mTOR, NLRP3-mediated caspase1/IL-1β, HIF-α, IGF1R, etc.

## 6. Conclusion and Future Prospects

The rising incidence of epilepsy cases, combined with the limited efficacy of the available anti-epileptic drugs, affects a significant number of people worldwide each year, hurting personal, social, and economic aspects of their lives. Adopting a targeted approach to combating both the disease-promoting risk factors and the pathomolecular processes that drive the development and progression of epilepsy may be more effective in reducing the symptoms associated with this neurological disorder. Considering that epileptic seizures are accompanied by a heightened central and systemic inflammatory response, inflammation may be a potential therapeutic target for the management of epilepsy. In addition, it is now known that neuroinflammation, or increased brain inflammation, interacts with various neurological components and processes associated with the pathophysiology of epilepsy.

In this review paper, we present the preclinical research findings that used flavonoids to target neuroinflammatory pathways in epilepsy. Thus, the review provides insight into a promising therapy for managing epilepsy by targeting neuroinflammation. Preclinical findings showed that flavonoids could reduce the production of pro-inflammatory cytokines by directly affecting inflammatory regulators like NF-kB and NLRP3 inflammasomes and altering the activity of resident immune cells in the brain, along with the proteins and signaling pathways that affect their activation. In these studies, flavonoids also prevented a long-term neuroinflammatory response by increasing the levels of anti-inflammatory cytokines and decreasing the levels of pro-inflammatory mediators such as COX-2, NOS, and others. Neuroinflammatory pathways are linked to the process of epileptogenesis, and flavonoids could be a promising therapy for treating epilepsy, given their considerable anti-neuroinflammatory potential. Despite the substantial number of preclinical studies showing that the anti-epileptic effects of flavonoids are strongly linked to the regulation of neuroinflammatory pathways, epilepsy patients are rarely examined for their anti-neuroinflammatory potential. To prove flavonoids’ effectiveness in treating epilepsy and comprehend entirely their anti-neuroinflammatory modes of action, which affect the molecular pathways regulating neuronal homeostasis in epilepsy, thorough preclinical and clinical investigations must yet be done.

## Figures and Tables

**Table 1:** Summary of anti-inflammatory properties of flavonoids in epilepsy.

Flavonoid	Model Used	Doses	Effects	References
Baicalein	Pilo-induced epilepsy in rats	40 mg/kg, i.p.	Reduces pro-inflammatory cytokines levels (TNF-α and IL-1β)	[82]
	Pilo-induced epilepsy in rats	20, 40, and 80 mg/kg, orally	Downregulates the expression of cytokines (IL-1β, IL-6, and TNFα), and IGF1R	[83]
	Tremor rats	10, 20, and 40 mg/kg, i.p.	Suppresses release of cytokines (TNF-α, IL-1β, IL-6, and IL-10), and p-JNK and p-p38 levels, while increasing pERK level	[84]
	PTZ-kindled rats	50 and 100 mg/kg, orally	Decreases levels of IL-1β and IL-6 and inhibits TLR4/MYD88/Caspase-3 pathway	[85]
Luteolin	KA-injected rats	10 and 50 mg/kg, i.p.	Suppresses microglial activation in hippocampus	[86]
	PTZ-induced seizures in rats	10 mg/kg, i.p.	Reduces iNOS and MMP-2 activity, and increases eNOS activity	[87]
Hispidulin	KA-induced seizures in rats	10 and 50 mg/kg, i.p.	Suppresses microglialosis, pro-inflammatory cytokines production (IL-1β, IL-6, and TNF-α), c-Fos expression and MAPK activation	[88]
Schaftoside	PTZ-induced seizures in zebrafish	100, 200, 400 μM	Decreases IL-1β, IL-6, NF-кB, and c-fos expression	[89]
Vitexin	Neonatal hypoxic ischemia-induced seizures in rats	45 mg/kg, i.p.	Decreases neutrophil infiltration and IL-1β, IL-6, and TNF-α expression	[90]
Wogonin	KA-induced TLE in rats	100 mg/kg, orally	Decreases IL-1β, TNF-α, and NF-kB expression	[91]
Rhoifolin	Hippocampal neuronal cell culture (HT-22 cell line)	5, 10, and 20 µM	Reduces IL-1β, IL-6 and TNF-α levels, and inhibits NF-кB/iNOS/COX-2 pathway	[92]
Amentoflavone	PTZ-kindled mice	25 mg/kg, orally	Decreases IL-1β, IL 18, and TNF-α expression, and inhibits NLRP3 inflammasome activation	[93]
	Pilo-kindled mice	25 mg/kg, orally	Decreases NO, PGE2, IL-1β and IL-6 production, inhibits NF-кB p65 activation	[94]
Quercetin	Febrile seizures in prenatally stressed rats	10 mg/kg, i.p.	Decreases levels of IL-1β, IL-6, and TNF-α	[95]
	KA-induced seizures in mice	100 mg/kg, i.p.	Reduces microglial activation, and levels of TNF-α, IL-1β, and activates NF-кB	[96]
	PTZ-kindled mice	25 and 50 mg/kg, i.p.	Reduces astrocytes activation	[97]
	Neonatal hypoxic ischemia-induced seizures in rats	25, 50, and 100 mg/kg, i.p.	Reduces IL-1β, IL-6, TNF-α, MCP-1 and iNOS levels and TLR4/NF-кB signaling in hippocampus	[98]
	PTZ-kindled rats	100 mg/kg, orally	Suppresses TNF-α, IL-6, IL-1β and NF-kB expression, and increases IL1Ra, IL-4, and IL-10 expression	[99]
Rutin	KA-kindled rats	50 and 100 mg/kg, orally	Suppresses astrocytes activation, downregulates IL-1β, IL-6, TNF-α, HMGB1, IL-1R1, and TLR-4 expression, and upregulates IL-10 expression	[100]
Fisetin	PTZ-kindled mice	5, 10, and 20 mg/kg, orally	Decreases HMGB1, TLR-4, IL-1R1, IL-1β, IL-6, and TNF-a levels, and NF-kB and COX-2 expression	[101]
Kaempferol	PTZ-kindled rats	100 mg/kg, orally	Downregulates TNF-α, IL-6, IL-1β and NF-kB expression and upregulates IL1Ra, IL-4, and IL-10 expression	[99]
Morin	PTZ-kindled rats	10 mg/kg, i.p.	Suppresses TNF-α expression, mitigates astrocyte activation, and IL-6/p-JAK-2/p-STAT3 signaling	[102]
	KA-kindled mice	20, 40, and 80 mg/kg, orally	Decreases microglial activation and IL-1β, TNF-α, and iNOS levels and inhibits mTORC1 pathway	[103]
Myricetin	PTZ-kindled mice	100 and 200 mg/kg, orally	Downregulate MMP-9 expression	[104]
Myricitrin	KA-induced TLE in rats	5 mg/kg, i.p.	Decreases TNF-α concentration	[105]
Galangin	PTZ-kindled mice	30 mg/kg, i.p.	Decreases microglial and astrocytic activation	[106]
Naringin	PTZ-kindled rats	20, 40, and 80 mg/kg, i.p.	Reduces TNF-α levels	[107]
	KA-kindled mice	80 mg/kg, i.p.	Decreases TNF-α expression in activated microglial cells	[108]
	KA-induced status epilepticus in rats	20, 40, and 80 mg/kg, i.p.	Decreases TNF-α expression	[109]
Naringenin	KA-kindled mice	50 and100 mg/kg, i.p.	Reduces IL-1β, and TNF-α levels in microglial cells and inhibits mTORC1 pathway	[110]
Hesperetin	KA-induced TLE in mice	5, 10, and 20 mg/kg, orally	Reduces TNFα, IL-1β, and iNOS levels	[111]
Hesperidin	Febrile seizure in rat pups	100 mg/kg, orally (Maternal administration)	Decreases TNF-α, IL 10, and TLR4 expression	[112]
	PTZ-induced seizures in Zebrafish larvae	1, 5, and 10 μM (Preincubated)	Reduces c-fos and IL-10 expression	[113]
Silibinin	Lithium-Pilo-induced TLE in rats	50 and 100 mg/kg, orally	Inhibits TNF-α, IL-1β, IL-6, and HIF-1α expression	[114]
	KA-kindled mice	50, 100, 200 mg/kg, i.p.	Inhibits TNF-α and IL-1β expressions and mTORC1 pathway	[115]
Genistein	PTZ-kindled rats	5 and 15 mg/kg, i.p.	Reduces astrocytes and microglial activation, and TNF-α, IL-1β, p-JAK2, p-STAT3 expression	[116]
Catechin	PTZ-kindled rats	100 mg/kg, orally	Suppresses TNF-α, IL-6, and IL-1β levels, and NF-kB 4 expression, and upregulates IL1Ra, IL-4, and IL-10 expression	[99]
Epigallocatechin-3-gallate	Lithium-Pilo-induced TLE in rats	25 mg/kg, i.p.	Decreases TLR4, NF-kB, and IL-1β expression	[117]
	KA-induced kindling TLE in mice	30 mg/kg, i.p.	Inhibits astrocytes and microglial activation	[118]

## Data Availability

Data sharing not applicable-no new data generated.
